# The Feasibility and Utility of Harnessing Digital Health to Understand Clinical Trajectories in Medication Treatment for Opioid Use Disorder: D-TECT Study Design and Methodological Considerations

**DOI:** 10.3389/fpsyt.2022.871916

**Published:** 2022-04-29

**Authors:** Lisa A. Marsch, Ching-Hua Chen, Sara R. Adams, Asma Asyyed, Monique B. Does, Saeed Hassanpour, Emily Hichborn, Melanie Jackson-Morris, Nicholas C. Jacobson, Heather K. Jones, David Kotz, Chantal A. Lambert-Harris, Zhiguo Li, Bethany McLeman, Varun Mishra, Catherine Stanger, Geetha Subramaniam, Weiyi Wu, Cynthia I. Campbell

**Affiliations:** ^1^Center for Technology and Behavioral Health, Geisel School of Medicine, Dartmouth College, Lebanon, NH, United States; ^2^Center for Computational Health, International Business Machines (IBM) Research, Yorktown Heights, NY, United States; ^3^Division of Research Kaiser Permanente Northern California, Oakland, CA, United States; ^4^The Permanente Medical Group, Northern California, Addiction Medicine and Recovery Services, Oakland, CA, United States; ^5^Department of Biomedical Data Science, Geisel School of Medicine, Dartmouth College, Lebanon, NH, United States; ^6^Department of Computer Science, Dartmouth College, Hanover, NH, United States; ^7^Khoury College of Computer Sciences, Northeastern University, Boston, MA, United States; ^8^Department of Health Sciences, Bouvé College of Health Sciences, Northeastern University, Boston, MA, United States; ^9^Center for Clinical Trials Network, National Institute on Drug Abuse, Bethesda, MD, United States; ^10^Department of Psychiatry and Behavioral Sciences, University of California, San Francisco, San Francisco, CA, United States

**Keywords:** opioid use disorder (OUD), digital phenotyping, medication for opioid use disorder (MOUD), ecological momentary assessment (EMA), passive sensing, social media

## Abstract

**Introduction:**

Across the U.S., the prevalence of opioid use disorder (OUD) and the rates of opioid overdoses have risen precipitously in recent years. Several effective medications for OUD (MOUD) exist and have been shown to be life-saving. A large volume of research has identified a confluence of factors that predict attrition and continued substance use during substance use disorder treatment. However, much of this literature has examined a small set of potential moderators or mediators of outcomes in MOUD treatment and may lead to over-simplified accounts of treatment non-adherence. Digital health methodologies offer great promise for capturing intensive, longitudinal ecologically-valid data from individuals in MOUD treatment to extend our understanding of factors that impact treatment engagement and outcomes.

**Methods:**

This paper describes the protocol (including the study design and methodological considerations) from a novel study supported by the National Drug Abuse Treatment Clinical Trials Network at the National Institute on Drug Abuse (NIDA). This study (D-TECT) primarily seeks to evaluate the feasibility of collecting ecological momentary assessment (EMA), smartphone and smartwatch sensor data, and social media data among patients in outpatient MOUD treatment. It secondarily seeks to examine the utility of EMA, digital sensing, and social media data (separately and compared to one another) in predicting MOUD treatment retention, opioid use events, and medication adherence [as captured in electronic health records (EHR) and EMA data]. To our knowledge, this is the first project to include all three sources of digitally derived data (EMA, digital sensing, and social media) in understanding the clinical trajectories of patients in MOUD treatment. These multiple data streams will allow us to understand the relative and combined utility of collecting digital data from these diverse data sources. The inclusion of EHR data allows us to focus on the utility of digital health data in predicting objectively measured clinical outcomes.

**Discussion:**

Results may be useful in elucidating novel relations between digital data sources and OUD treatment outcomes. It may also inform approaches to enhancing outcomes measurement in clinical trials by allowing for the assessment of dynamic interactions between individuals' daily lives and their MOUD treatment response.

**Clinical Trial Registration:**

Identifier: NCT04535583.

## Introduction

Across the U.S., the prevalence of opioid use disorder (OUD) and the rates of opioid overdoses have risen precipitously in recent years. Drug overdose has been called a “modern plague” (1) and is the leading cause of death of Americans under age 50, having surpassed peak death rates from gun violence, HIV, and car crashes (1, 2). Over 100,000 Americans died from a drug overdose from May 2020 to April 2021 (3). This dramatic spike in OUD has also been accompanied by marked increases in injection-related infections (including infective endocarditis and Hepatitis C) (4–7), babies born with Neonatal Opioid Withdrawal Syndrome (8) and healthcare and criminal justice costs (9).

Several effective medications for OUD (MOUD) have been shown to be life-saving including buprenorphine, methadone, and naltrexone products (10, 11), and to greatly increase opioid abstinence, reduce HIV/infectious disease risk behavior, and reduce criminality. Greater MOUD retention is associated with the most positive treatment outcomes (12–16). However, over 50% of patients receiving MOUD dropout of treatment within 3–6 months after treatment initiation (17–21), falling short of the longer threshold of treatment shown to offer sustained benefit (22, 23). Additionally, given the chronic relapsing nature of the disease of addiction, and inconsistent compliance with MOUD, many individuals continue to engage in opioid use during treatment, increasing the risk of overdose (24, 25).

Many factors predict attrition and continued substance use during substance use disorder (SUD) treatment (26, 27), including, stress, mental health comorbidities, continued exposure to high-risk social networks or contexts, and the neurobiology underpinning addiction. However, this literature has examined a limited set of predictors of outcomes in MOUD treatment and may not reflect a comprehensive understanding of treatment non-adherence (28). Further, treatment engagement is typically evaluated via structured clinical assessments conducted on an episodic basis and may not reflect factors in individuals' daily lives that impact their OUD treatment trajectories. Thus, there is tremendous opportunity to more frequently and extensively examine factors that impact individuals' clinical trajectories in MOUD in real time.

Digital methodologies offer great promise for capturing intensive, longitudinal ecologically-valid data from individuals receiving MOUD to extend our understanding of factors that impact treatment engagement and outcomes (29, 30). In particular, the use of digital devices such as smartphones or wearables that measure individuals' health-related behavior (sometimes referred to as “digital phenotyping”) (31) has the potential to provide personalized health care resources. The ubiquity of digital devices and the explosion of “big data” analytics enable the collection and interpretation of enormous amounts of rich data about everyday behavior. This includes the use of digital devices to implement “ecological momentary assessment” (EMA) (32) in which individuals are asked to respond to brief queries on their mobile devices (assessing, for example, craving, mood, withdrawal symptoms, and pain). It also includes passive sensing data collected via sensors embedded in smartphones and/or wearable sensing devices such as smartwatches that provide information about the wearer's health (e.g., heart rate and heart-rate variability measured via wearable photoplethysmography), behavior (e.g., social contact via calls, texts and app use), and environment (e.g., location type via GPS) (33). And, it includes social media data that individuals produce (e.g., the images and the texts they post).

A rapidly growing literature is underscoring the utility of such digital health data-driven approaches to understanding human behavior (34–37). Digitally-derived data may similarly reveal new insights into the temporal dynamics between moderators and mediators of MOUD treatment outcomes. Such data may complement and extend data captured via structured clinical assessments and provide a more comprehensive understanding of each individual's course of treatment. And these data, in turn, may increase our ability to develop more potent and personalized treatment models for OUD.

The developing literature on the application of digital health to understanding individuals' trajectories in SUD treatment has shown promise. One study that used EMA to identify predictors of substance use among adults after an initial episode of SUD treatment showed high EMA completion rates (81%) and identified specific substance use patterns, negative affect and craving as predictors of substance use (38). EMA research has also demonstrated differing relationships between drug triggers (e.g., exposure to drug cues or mood changes) and different types of drug use. Specifically, drug triggers increased for hours before cocaine use events but not before heroin use events (39). And, among smokers trying to quit, smoking lapses were associated with increases in negative mood for many days (and not just hours) (40).

Additionally, EMA research with adults in MOUD treatment demonstrated a stronger relationship between craving and drug use events than between stress and drug use events (41). EMA-assessed momentary pain has been shown to be indirectly associated with illicit opioid use via momentary opioid craving (42). Further, MOUD treatment dropout has been shown to be more likely among individuals who report more “hassles”, higher levels of cocaine craving, lower levels of positive mood, a recent history of emotional abuse, and a recent history of being bothered frequently by psychological problems. It is noteworthy that none of those factors predicted individuals' non-compliance with completing EMA (43). Other EMA research revealed that patients in MOUD treatment who share similar patterns of drug use (frequent opioid use, frequent cocaine use, frequent dual use of opioids and cocaine, sporadic drug use, or infrequent drug use) tended to have similar psychological processes preceding drug use events (44).

Less research has focused on the utility of passively collected sensing data or social media data in predicting substance use. One study used GPS data from phones to assess exposure to visible signs of environmental disorder and poverty among adults in outpatient MOUD treatment. That study provided a proof of concept that digitally-captured environmental data could predict drug craving and stress 90 min into the future (45). Another study with adults in outpatient MOUD, focused on passive assessment of stress, showed that the duration of a prior stress episode predicts the duration of the next stress episode and that stress in the mornings and evenings is lower than during the day (46). And another study demonstrated that deep-learning analytic approaches applied to social media data may be useful in identifying potential substance use risk behavior, such as alcohol use (47).

Overall, these findings have provided some new insights into how data collected in naturalistic settings may enhance an understanding of risk profiles among individuals in SUD treatment. Nonetheless, the breadth of factors evaluated to date has been limited, and most digital health studies conducted with populations in SUD treatment have relied exclusively on self-reported clinical outcomes (with limited focus on objective metrics such as urine screens, medication fills, and clinical visits). Additionally, most studies have solely sought to predict substance use events.

This paper describes the protocol (including the study design and methodological considerations) from a novel study supported by the National Drug Abuse Treatment Clinical Trials Network (CTN) at the National Institute on Drug Abuse (NIDA). This study, referred to as “Harnessing Digital Health to Understand Clinical Trajectories of Opioid Use Disorder” (D-TECT; CTN-0084-A2) primarily seeks to evaluate the feasibility of collecting EMA, digital sensing and social media data among patients in outpatient MOUD treatment. It secondarily seeks to examine the utility of EMA, digital sensing, and social media data (separately and compared to one another) in predicting MOUD treatment retention, opioid use events, and medication adherence [as captured in Electronic Health Records (EHR), medical claims, and EMA data]. This is the first project to include all three sources of digitally derived data (EMA, sensing and social media) in understanding the clinical trajectories of patients in MOUD treatment. Multiple data streams will allow us to understand the relative and combined utility of collecting digital data from these diverse data sources. The inclusion of EHR data allows us to focus on the utility of digital health data in predicting objectively measured clinical outcomes.

## Methods and Analysis

### Overview of Study Design

**Figure 1:**
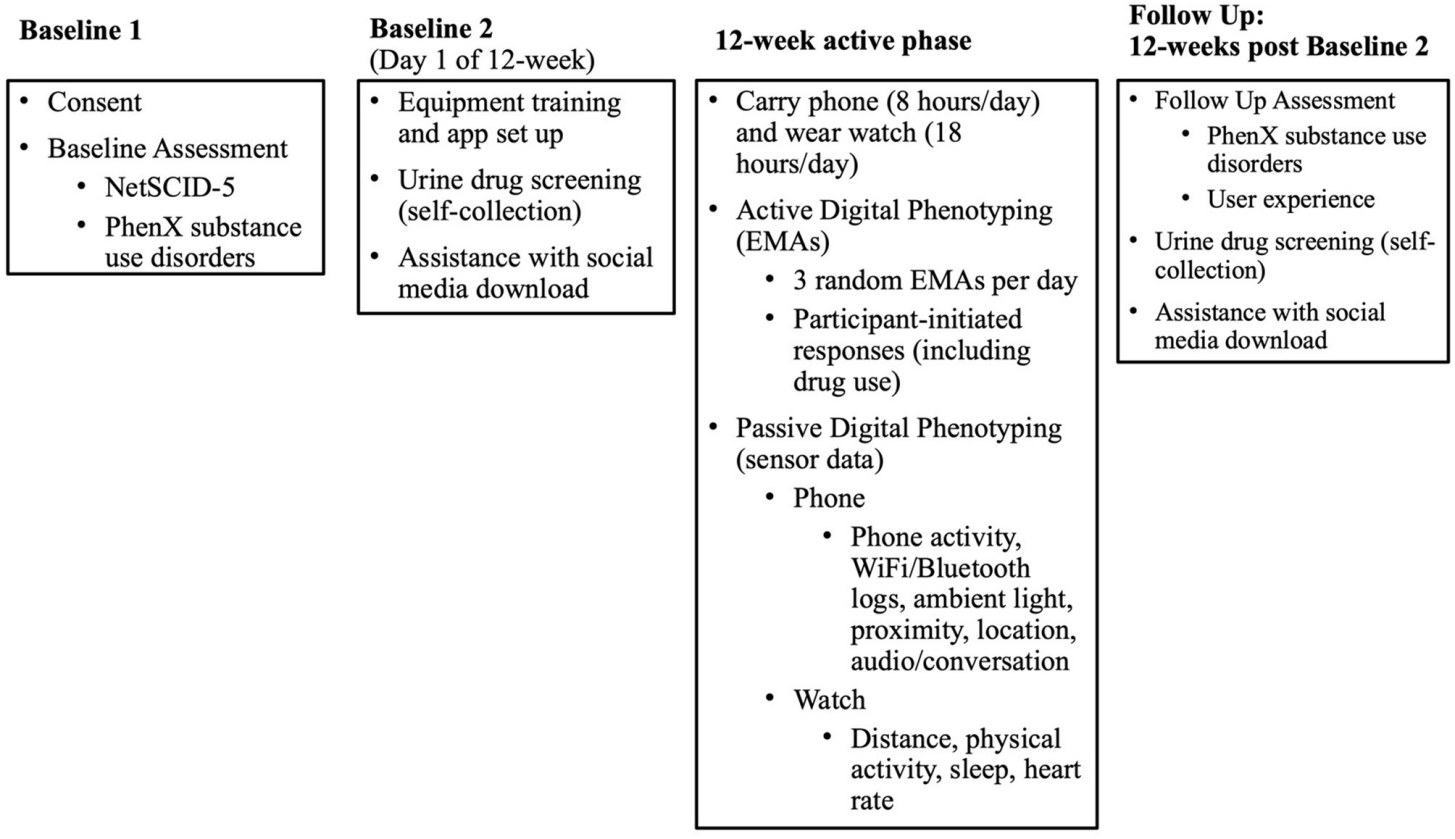
Study phases.

Individuals with OUD will be recruited for the study from among patients who are active in outpatient MOUD treatment with buprenorphine medication for at least 2 weeks at one of four Addiction Medicine Recovery Services (AMRS) programs at Kaiser Permanente Northern California (KPNC). Once it is confirmed that eligibility criteria are met, each participant will provide electronic informed consent and complete the baseline assessment by phone. The baseline appointments will take ~2.0 h to complete, which will be done in two to three visits. Participants will be asked to wear a smartwatch and carry a smartphone (a study-supplied one or their own) that will passively collect sensor data. They will be asked to actively respond to EMA prompts through a smartphone 3 times daily and to self-initiate EMA responses daily if substance use occurred over the 12-week study. For those who consent to the optional social media component, social media data will be downloaded by the participant directly from the social media platform to a secure server using a remote desktop at the beginning of the study and again at the end of the study. EHR data extraction will occur at ~16 weeks after the full study is completed and will collect data 12 months prior to EMA start (the date the participant began receiving EMA prompts) through 12-weeks after EMA start (84 days after the EMA start date). A follow-up assessment (~45 min in length) will occur by phone ~12 weeks after EMA start. A graphic overview of the study phases is presented in Figure 1.

### Study Sites and Rationale for Site Selection

KPNC is a large, integrated health care delivery system with 4.3 million members, providing care through commercial plans, Medicare, Medicaid, and health insurance exchanges. It is comprised of a racially and socioeconomically diverse membership and is generally representative of the region's population with access to care. KPNC was selected based on its ability to (1) provide access to individuals who are prescribed buprenorphine for OUD and (2) provide access to EHR data on treatment retention, medication adherence, and service utilization. KPNC maintains a data repository, the Virtual Data Warehouse, which has combined EHR data (e.g., demographics, membership, diagnoses, service utilization, pharmacy, lab data) with several other data sources, including medical claims data (e.g., non-Kaiser pharmacy data).

The AMRS programs at KPNC offer a broad range of services, including prescribing buprenorphine for OUD, medical services, group and individual therapy, and family therapy. Staffing includes physicians, therapists, medical assistants, nurses, and social workers.

### Participants

Participants will include individuals aged 18 years or older across all racial and ethnic categories. Eligibility criteria include: active in KPNC outpatient treatment and prescribed buprenorphine for OUD for the past 2 weeks (and attended at least one visit at AMRS in past 35 days); >18 years old; capable of understanding and speaking English; able to participate in the full duration of the study (12 weeks); have an active email account and willing to provide its address to researchers; permit access to EHR data; willing to carry and use a personal or study-provided smartphone for 12 weeks; and willing to wear a smartwatch continuously (except during pre-defined activities such as showering) for 12 weeks. Individuals will be excluded if they are: unwilling or unable to provide informed consent; currently in jail, prison or other overnight facility as required by court of law or have pending legal action that could prevent participation in study activities. We expect to recruit 50–75 participants.

### Study Procedures

#### Recruitment and Screening

Potentially eligible patients will be initially identified from EHR data as meeting study criteria; eligibility is further confirmed through chart review. Eligible patients will be sent an invitational recruitment letter through a secure email message. Within approximately a week of mailing recruitment letters (or sending a secure email message), research staff will contact participants by phone to determine if they are interested and eligible, using the IRB-approved recruitment script, verbal consent form and final screening questions. If the individual is interested and eligible, research staff will schedule them for an initial baseline phone appointment and email them consent documents for their review before their appointment.

#### Informed Consent

Informed consent will be obtained by phone and documented online using an electronic signature. Each participant will be asked to pass a brief consent quiz to document comprehension of the study activities. The research staff will obtain authorization from participants for use of protected health information, such as their EHR and medical claims data.

#### Baseline

The baseline process will be conducted in two or three phone appointments: the first appointment will consist of informed consent and the baseline assessment (Baseline 1), and the second (and third, if necessary) appointment (Baseline 2/3) will consist of a urine drug screen, setting up study devices, installing study applications (“apps”), and learning to use devices and apps (Figure 1).

The baseline assessment consists of interviewer-administered measures (described below) examining participant characteristics, current substance use (e.g., tobacco, alcohol, opioids, and other drugs), substance use and mental health disorders, and the impact of the COVID-19 pandemic. Once the first baseline appointment is completed, participants will be mailed a urine drug screen kit, a smartwatch and study smartphone (if applicable) and technology training documentation.

Once the equipment is received, research staff will schedule a second phone appointment with each participant to review the urine collection and technology training documentation. Research staff will walk through the set-up, use and care of the smartphone and smartwatch, installation of the study app and the Garmin Connect app (described below) if they are not already installed on a study-provided phone, as well as instructions for initiating and completing the daily EMA surveys. Research staff will also instruct participants to collect a urine sample and upload results.

Participants who consented to the social media part of the study will also receive instruction on how to request and download their social media data.

#### Active Study Phase (12-Weeks)

During the active 12-week study phase, the research staff will monitor participant compliance using a custom dashboard (e.g., their EMA completion rate and whether they carry the study phone and wear the study watch). In the first 2 weeks, participants will be followed closely. If after a 48-h period there are no EMA data, and/or no phone carry time data, and/or no watch wear data, research staff will follow-up directly with the participant via phone, text, and/or email to encourage the participant to continue their participation and/or troubleshoot any problems that may arise with the smartphone, smartwatch, and/or study app. All participants will have a 1-week check-in appointment via phone with research staff to review the participant's experience, review data collection over the past week, and answer any questions or resolve any technical issues with the study devices (regardless of device carry/wear time compliance or EMA completion rate). Thereafter, research staff will send weekly check-in texts or phone calls unless there is a 48-h period of no EMA data, and/or no phone carry time data, and/or no watch data. In those instances, the research staff will attempt to reach the participant by phone, text, and/or email. The research staff will make up to three contact attempts prior to engaging alternate contact(s).

#### Engagement in Research

To be considered engaged in the study, an individual must respond to a minimum number of EMA prompts (complete at least 2/3 of the EMA surveys per day on 7 out of the first 14 days) and record at least 8 h of smartphone/smartwatch sensor data per day on 7 out of the first 14 days of study participation. If an individual does not meet the engagement criterion and is non-responsive to research staff outreach in the first 14 days of study participation, then the individual will be considered a “non-engager” and the study team will continue to recruit until the targeted sample size is met. Non-engagers will not be withdrawn from the study, as we will attempt to collect all possible data from all participants.

#### Follow-Up

A follow-up assessment will be completed by phone ~12 weeks post-EMA start. Research staff will administer an interviewer-based assessment to measure current substance use, participant experience with the study devices, treatment utilization, reasons for drop out (if appropriate), employment, insurance coverage, medication use/dose (if applicable), and overdose (if applicable). Participants will be mailed a urine drug test kit and asked to collect another urine sample and upload test results. Participants who consented to the social media part of the study will be asked to request and download their social media data a second time.

### Description of Measures

**Figure 2:**
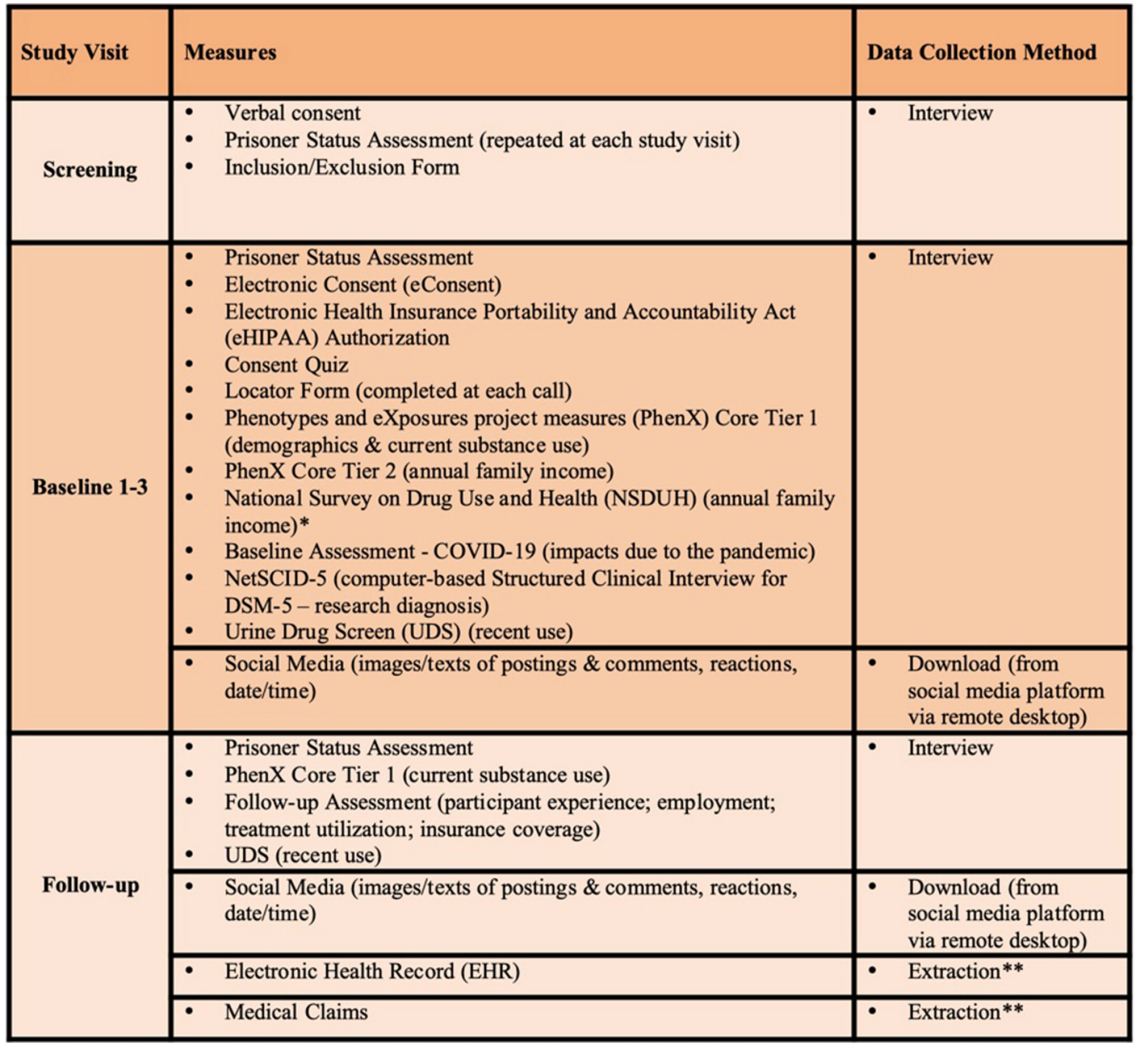
Table of study assessments. *NSDUH is only collected for participants who are unsure of the total family income. We will use as subset of questions to determine which income category best characterizes total combined family income. **EHR/Medical Claims data will be extracted by the data analyst ~16 weeks after completion of study and includes data 12 months prior to EMA start through 12 weeks post-EMA start.

**Figure 3:**
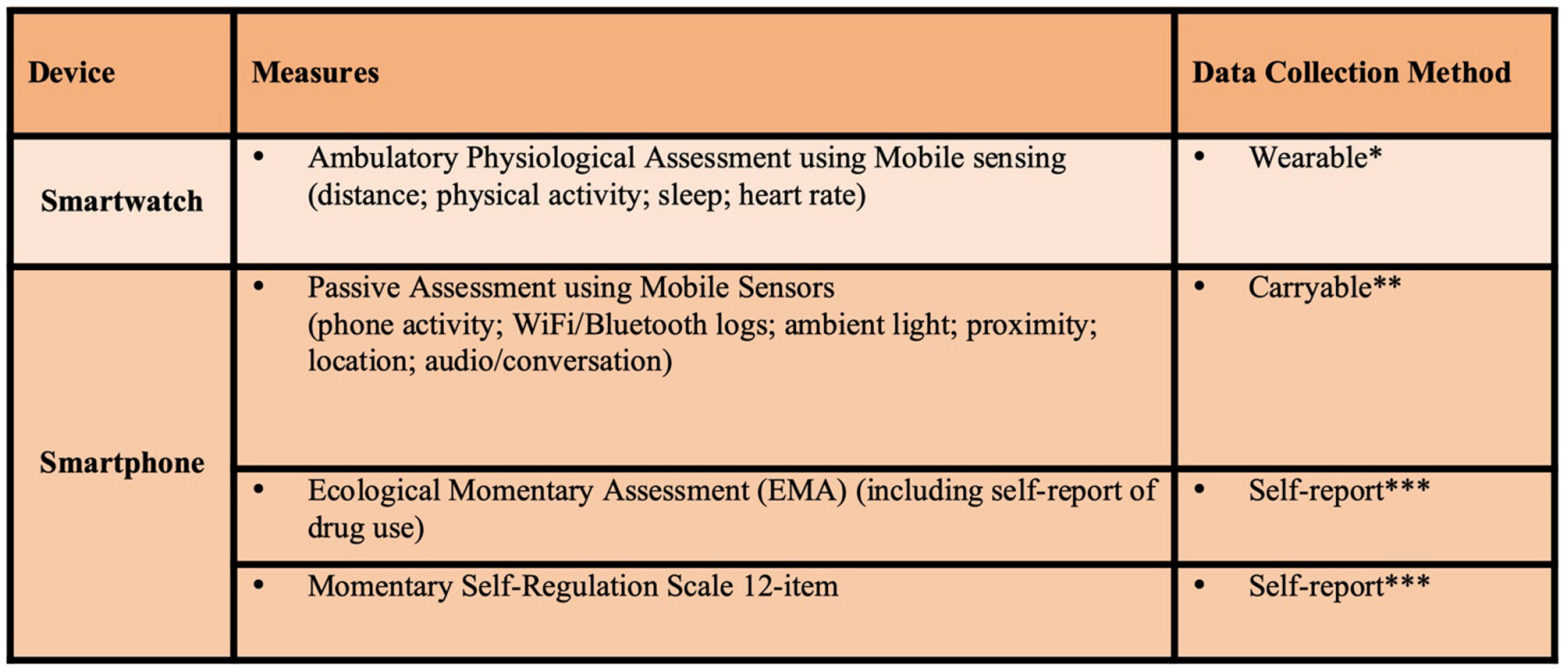
Active study phase digital health assessments. *Wear smartwatch at least 18 hours per day, data transmitted real-time for 12 weeks. **Carry smartphone at least 8 hours per day, data transmitted real-time for 12 weeks. ***3 times per day for 12 weeks.

A summary of the clinical assessments and digital health assessments to be conducted in this study are reflected in Figures 2, 3, respectively. Brief descriptions of each of these measures is provided below.

**Prisoner Status Assessment:** An individual's prisoner status must be assessed for each participant at each separate encounter, as this study will not apply for Office of Human Research Protection (OHRP) Prisoner Certification. An **Inclusion/Exclusion** form will be used to obtain information on inclusion and exclusion criteria to document eligibility. **Locator Form**. A locator form is used to obtain information at baseline and each contact to assist in finding participants throughout the study. This form collects the participant's current address, email address, and phone numbers. **PhenX Substance Abuse and Addiction Core Tier 1 (PhenX Core Tier 1)**. The PhenX Core Tier 1 is a part of the Substance Abuse and Addiction Collections (48) that are being adopted across multiple studies funded by NIDA. This study will use the following subset of measures from the Core Tier 1: demographics (age, ethnicity, gender, race, current educational attainment, current employment status, and current marital status) and current substance use (tobacco, alcohol, and drugs) (49). The demographics (except for employment status) will only be collected at baseline, while current substance use and current employment status will be collected at baseline and follow-up. **The PhenX Substance Abuse and Addiction Core Tier 2** (PhenX Core Tier 2). The PhenX Core Tier 2 is a complementary set of 8 measures to the PhenX Core Tier 1 (i.e., annual family income, child-reported parental education attainment, family history of substance use problems, household roster-relationships, internalizing, externalizing, and substance use disorders screener, occupation/occupational history, peer/partner substance use and tolerance of substance use, and social networks) (50). This study will only use a subset of questions from the Annual Family Income measure to get an estimate of total income of all family members (49). If the participant is unsure of the total family income, then we will use a subset of questions from the Substance Abuse and Mental Health Services Administration's National Survey on Drug Use and Health (NSDUH) survey to determine which income category best characterizes total combined family income (51). These measures will be securely, electronically stored in a REDCap database.

**NetSCID-5:** The Structured Clinical Interview for DSM-5 (SCID-5) is a semi-structured interview designed to assess substance use and mental health diagnoses (52). This study will use an electronic version of the SCID-5, the NetSCID-5, developed by TeleSage. The TeleSage NetSCID-5 is fully licensed by the American Psychiatric Association and has been validated (53). TeleSage has the capability of customizing the NetSCID-5 measure, and modules relevant for this study include: bipolar I disorder, major depressive disorder, panic disorder, social anxiety disorder, generalized anxiety disorder, posttraumatic stress disorder, adult attention deficit hyperactivity disorder, alcohol use disorder, and other use disorders [cannabis, stimulants/cocaine, opioids, phenylcyclohexyl piperidine (PCP), other hallucinogens, inhalants, sedative-hypnotic-anxiolytic, and other/unknown]. The TeleSage NetSCID-5 will be administered by research staff who are trained and credentialed to conduct this diagnostic assessment.

**Urine Drug Screen:** Urine drug screen kits will be mailed to participants, and participants will be asked to collect a urine sample and then record and upload its results using a secure system (e.g., REDCap) at first baseline appointment and at follow-up. All urine specimens are collected using CLIA-Waived and FDA-approved one-step multi-drug screen test cups following the manufacturer's recommended procedures. The study will use the DrugConfirm Advance Urine Drug Test Kit that screens for: alcohol, amphetamine, barbiturate, buprenorphine, benzodiazepine, cocaine, fentanyl, MDMA (ecstasy/molly), methamphetamine, methadone, morphine 300 ng/mL, oxycodone, phencyclidine (PCP), tramadol and delta-9-tetrahydrocannabinol (THC).

In order to reduce risks of substituted or adulterated urine samples, the research staff will conduct the study urine drug screens in real-time (i.e., reviewing collection instructions and walking the participant through the entire process via phone) during the Baseline 2 phone appointment. Participants will send a photo of the temperature strip to research staff immediately after they produce the sample to ensure the temperature is within the specified valid temperature range of 90°-100°F. Additionally, we will ask participants take photos of the test result strips and send the photos of the results securely to research staff in real-time (while the participant is still on the phone). Research staff will review the photos that are sent to ensure that the results captured within the photos are legible and not blurry or otherwise indecipherable.

### Digital Health Technology

#### Ambulatory Physiological Assessment Using Mobile Sensors

We will develop a smartphone application (“study app”) for both Android and iOS devices. The study app can sense and store contextual information about a participant, e.g., location, physical activity (step count), conversation duration and count (non-identified audio information such as segments of silence, and speech features such as pitch control and voice quality), app usage, call/text, screen on/off, phone lock/unlock, and phone notifications (54). Features will be derived from the raw sensor streams to create multiple relevant contextual variables. This custom application will be installed directly on the study-provided smartphone (Moto G7 Power and/or Moto G Power) or on a participant's smartphone if they have a compatible phone (iPhones, running iOS 12 or higher or Android devices running Android 8.0 or higher with at least 2.5 GB RAM and 4 GB of available storage).

In addition to the smartphone, participants will be provided with a smartwatch (Garmin Vivosmart 4). The participants will be asked to wear the smartwatch continuously (except during pre-determined exception periods, such as when the participants are showering or charging the device). The Garmin Vivosmart 4 smartwatch is comfortable, lightweight, and has a long battery life of up to 7 days (55) and an easy-to-use interface. The device can continuously collect and track a variety of sensor data in the background, as long as the user is wearing the device. The data from the wearable is synced directly with Garmin cloud servers, using the “Garmin Connect” application installed on the phone, and we will not have direct access to the raw sensor data. We will use the Garmin Health Connect API to get various health metrics that are computed by Garmin's proprietary algorithm, such as heart rate, sleep stage information (i.e., periods and events of light/deep sleep), stress levels, and physical activity levels (including energy expenditure) and step counts). Through the Garmin Health Connect API, Garmin's servers will push the various metrics computed from the raw data to the storage servers at Dartmouth College.

#### Ecological Momentary Assessment

Participants will be prompted 3 times per day over 12 weeks by the smartphone app to self-report sleep, stress, pain severity, pain interference, pain catastrophizing, craving, withdrawal, substance use risk context, mood, context, substance use, self-regulation, and MOUD adherence (41, 56–59). The EMA prompt delivery times will be randomized within each of the prompt timeframes (e.g., morning, mid-day, end of day). In addition to prompted EMAs, participants will be asked to self-initiate EMA responses if substance use occurred (e.g., opioids, cocaine, or other stimulants). When determining the rate of completion of self-initiated reports of substance use, we will be able to cross-reference responses to the following question asked in the “End of the Day” EMA prompt (“Did you use any drugs at all today without reporting it?”) with participant's self-initiated substance use EMA data.

Additionally, participants will be asked to complete a **Momentary Self-Regulation Scale**^1^ via EMA. This brief 12-item questionnaire assesses self-regulation on a momentary basis as individuals move through their daily lives. This information will be collected 3 times daily over the 12-week study period by smartphone.

#### Social Media

Participants will be asked to request and then download their social media data (Facebook, Instagram, or Twitter) to a secure server using a remote desktop application. These three social media platforms provide the functionality for each user through their account setting to download their social media data as an aggregated structured file. After requesting a data download from a social media website, the participant will receive an email notification when the downloadable copy of the data has been created—typically in <48 h from the request. Once the social media data are ready to download, the participant will log into a remote computer located at Dartmouth College by using Microsoft Remote Desktop and will download their social media data to the secure research study computer. After completing the download, the participant will sign out of the remote computer and alert the research team. Images and text postings as well as date/time for each post will be extracted from the downloaded social media data. As noted elsewhere, participation in this part of the study is optional; participants can still participate in the study and decline to provide their social media data.

We will parse the JSON/JS files of the downloaded social media data to extract the information of interest, including posting date, text, and corresponding image paths on a local storage. We will aggregate the extracted data into a pickle file composed of different data dictionaries for text, posting dates, and local image paths. It is noteworthy that all the social media data from three variant platforms will be in the same format after processing. We plan to collapse and aggregate the data collected across the three social media platforms to reduce data sparsity and thereby increase the number of study days that are represented in the training and evaluation data sets.

#### EHR Data

EHR data extraction will include all outpatient and inpatient encounters, medications, procedures, and diagnoses for the 12 months prior to EMA start and the 12-week study period. In addition, we will extract lab results from urine drug screens, patient demographic information, KPNC health plan membership status, and insurance deductible level. We will extract appointment data to determine if visits were canceled or missed. KPNC is also an insurance plan and has claims data on non-KPNC services that were submitted as medical claims for reimbursement.

#### Clinical and Safety Assessments

Clinical and safety events may be elicited at baseline or spontaneously reported to study staff at any encounter following consent. Safety events suggesting medical or psychiatric deterioration will be brought to the attention of the study clinician for further evaluation and management.

### Compensation

Participants will be compensated up to $21 per week for completing EMA surveys, up to an additional $10 per week bonus for completing a minimum of 80% of received EMAs, and up to $14 per week for carrying their smartphone at least 8 h per day and wearing the smartwatch at least 18 h per day. At the end of the 12-week active phase of the study, participants will receive a $50 bonus for either using their personal smartphone or returning a study-provided smartphone, and a $50 bonus for returning the study-provided smartwatch. Finally, participants who consent to the social media portion of the project will receive up to an additional $180. Total possible compensation will be up to $820 over the course of the 12-week active study phase for the digital data collected (i.e., each EMA completed plus EMA bonus, smartphone carry time met and smartwatch wear time met, and social media data download, if applicable). In addition to the earnings and bonuses (described above), an individual who completes a minimum of 80% of received EMA surveys within a given week will qualify for a drawing at the end of that week where the individual could win a $50 prize. Each individual will have an opportunity to participate in up to 12 drawings over the 12 weeks. During the 12-week active study phase, any incentives, bonuses, and/or drawings earned will be uploaded weekly to a reloadable debit card. Study participants will be compensated $75 for completing the baseline appointments and baseline urine drug screen (via Target gift card), and $100 for completing the 12-week follow-up appointment and follow up urine drug screen (via Target gift card). Total compensation will be up to $995 for participating in all study activities [digital compensation ($820) plus baseline and follow-up appointment compensation ($175)].

### Statistical Analyses

#### Primary and Secondary Outcomes (Endpoints) and Hypotheses

The primary outcomes will include (1) the percentage of days during the 12-week active phase enrolled participants met criteria for wearing the smartwatch and carrying the smartphone; (2) the response rate to EMA prompts during the 12-week active phase; and (3) the percentage of participants who consent to social media data download and sparsity of social media data per participant. We hypothesize that the majority of participants who enroll in the study will wear the smartwatch, carry the smartphone, respond to EMA prompts, and be willing to share their social media data with the research team. We expect the number of participants deemed “non-engagers” will be low.

The secondary outcome measures will be (1) OUD treatment retention (days retained in OUD treatment program) based on EHR data; (2) days covered on MOUD based on EHR and EMA data; and (3) non-prescribed opioid use based on EHR and EMA data. We hypothesize that intensive longitudinal digital data capturing patient context and psychological state will be useful for predicting treatment retention, opioid use events and buprenorphine medication adherence.

#### Statistical Methods

For our primary feasibility assessment for primary and secondary outcomes, we will generate descriptive statistical summaries of the level of adherence of study participants to the desired protocol (e.g., EMA response rate, smartwatch wear rate and smartphone carry rate).

For our predictive analyses for primary and secondary outcomes, we are interested in measuring and predicting outcomes that may occur repeatedly over a 12-week observational period (e.g., patterns of daily drug use) using digital health technology. In digital health the spatio-temporal granularity of information about an individual is of higher resolution than that obtained through cross-sectional or traditional longitudinal studies (60). We will therefore assess the utility of using data from smartphones, smartwatches, social media, and ecological momentary assessment to predict, explain and detect these outcomes.

Our approach to prediction will include regression methods (e.g., logistic regression), but we will also use various machine-learning approaches for binary classification (e.g., random forest, support vector machines, K-means, gradient boosted trees, neural networks). For each of these classification techniques, we will assess the utility of the various digital data for improving prediction quality.

The study will generate the nested longitudinal data with binary response sequences collected over time. The regression model (logistic regression) will be built to account for the nested data structures by incorporating both fixed effects and random effects, which would allow us to examine both inter and intra-individual differences. Machine learning models can also be integrated with the random-effects structure as in the mixed-effect models (61). In the cross-validation, the training data will be split into k-folds by patient id. Previous work has shown that whether training data is split by record or by patient can significantly affect model performance (62), with better performance typically being achieved when splitting data by record rather than by participant.

For social media data, we will use deep neural networks for feature extraction and predictive analysis. Specifically, pretrained residual neural network (ResNet) (63) will be used to extract features from images and bidirectional encoder representations from transformers (BERT) (64) models will be used to extract features from text. Using these neural networks, social media images and text can be represented as dense vectors that can be aggregated with the rest of the collected data for predictive analysis. We will also explore classic machine-learning methods (such as random forest, support vector machines, and gradient boosted trees) for social media-based prediction, and compare their results to the performance of deep neural networks. Typical evaluation methods used to assess the prediction quality include area under the receiver operating characteristic curve (AUROC), accuracy, precision, F-score, sensitivity (recall), and specificity. The relative utility of the various data for predicting outcomes will be assessed at two levels - individual features and aggregated features (e.g., Facebook, Twitter, GPS, step, sleep, mood). The contribution of each feature in predicting the outcome variable will be assessed using a model-agnostic machine-learning approach to reverse-engineering algorithms by perturbing model inputs based on game theory, SHapley Additive exPlanations (SHAP) (65, 66).

When missing data are encountered, we will apply domain knowledge to reflect on the probable reasons that the data are missing. Based on our knowledge-based assessment of the nature of the missing data, missing samples will be imputed using appropriate imputation methods (67, 68).

There are at least two approaches to integrating data from the three data sources for use by a single prediction model, depending on whether the prediction models operate in a lower-dimensional “latent/embedding space” or a higher-dimensional “feature space”. Deep learning models typically operate in the latent/embedding space, while other machine learning models (e.g., Random Forest, Gradient Boosted Regression Tree) operate in the feature space.

When combining data in the feature space, features must be engineered from both structured and unstructured data. The unstructured social media data, in particular, may require manual or automated annotation in order to generate features. When combining data in the latent space, models that convert both structured and unstructured data into the latent space will be required. These models could be pre-trained on other similar data sources (e.g., BERT for natural language text, pre-trained ResNet model on ImageNet for image data, Activity2Vec for sensor data). We are not aware of pre-trained models for generating embeddings from EMA/survey data.

Yet another approach for “integrating” all 3 data sources is to train separate models using each dataset and an ensemble predictor that combines the predictions from each model to generate a final prediction, e.g., bagging or accuracy-weighted ensemble (69).

We will perform k-fold cross validation (CV) when evaluating the performance of the prediction models. We will do a group k-fold CV where instead of randomly splitting all data into k-folds, we will divide our dataset into k groups such that each participant is assigned to only one group with no overlap between the groups. This is to prevent any data leakage that might happen due to a participant's data being present in the train and test sets.

This is an exploratory pilot study. Therefore, a detailed analysis of statistical power to detect effects was not performed. As we are predicting daily outcomes (e.g., daily medication adherence, daily drug use), the sample size that is potentially available to us is equal to the number of participants multiplied by the number of study days. For example, assuming 60 participants in the study, and a study period of 12 weeks (i.e., 84 days), the analytic sample size would be 60^*^84 = 5,040 participant-days. If the observed incidence of non-adherence or drug use is 10%, then we would observe ~504 non-adherence or drug use events. Results of this study may generate a data set that could be helpful for future researchers to estimate the likely power of predictive models for this patient population, using similar sources of data.

## Discussion

In a world that is rapidly embracing digital health approaches to understand and provide resources to support health behavior, this study is distinct in that it will be the first to systematically assess the feasibility and utility of digitally-derived data from EMA, passive sensing and social media, all collected from the same sample of individuals in MOUD treatment. Results from this study may be useful in elucidating novel relations between digital data sources and treatment outcome. It may also inform approaches to enhancing outcomes measurement in clinical trials by allowing for the assessment of dynamic interactions between individuals' daily lives and their MOUD treatment response. It may additionally inform specific digital data collection protocols in the next phase of this line of research, including the need to abbreviate EMA questions to capture those most clinically useful and/or strategies for addressing any privacy or data sharing concerns that may arise among participants. As the opioid epidemic and opioid overdoses surge in the U.S., this novel study and its clinically-relevant implications are timely.

## Ethics Statement

The studies involving human participants were reviewed and approved by the Institutional Review Board (IRB) at Kaiser Permanente Northern California (the single IRB overseeing this study). The patients/participants provided their written informed consent to participate in this study. We plan to disseminate findings from this research at scientific meetings and in multiple peer-reviewed publications, including results of analyses assessing the feasibility of using EMA, digital sensing and social media data among adults in outpatient MOUD and analyses assessing the predictive utility of these data sources in predicting MOUD treatment retention, opioid use events, and medication adherence.

## Author Contributions

LM, CC, C-HC, SH, DK, CL-H, and CS were responsible for developing the study design for this project. LM wrote the initial draft of the protocol paper. AA, CC, MD, MJ-M, HJ, CL-H, and BM were involved in study coordination and operations. GS was the study's Scientific Officer. SA, CL-H, VM, and WW prepared the data sets for analyses. NJ consulted on the statistical analysis plan. C-HC, ZL, VM, and WW conducted the statistical analyses. EH prepared the references. All authors participated in the review and revision process and approved the submission of this version of the protocol paper.

## Funding

This work was supported by the National Institute on Drug Abuse (NIDA) Clinical Trials Network grant UG1DA040309 (Northeast Node), UG1DA040314 (Health Systems Node) and NIDA Center grant P30DA029926. NIDA had no role in the study design; in the collection, analysis, and interpretation of the data; in the writing of the report; or in the decision to submit the paper for publication.

## Author Disclaimer

This manuscript reflects the views of the authors and may not reflect the opinions, views, and official policy or position of the U.S. Department of Health and Human Services or any of its affiliated institutions or agencies.

## Conflict of Interest

C-HC and ZL are employed by IBM Research. CC has received support managed through her institution from the Industry PMR Consortium, a consortium of companies working together to conduct post-marketing studies required by the Food and Drug Administration that assess risks related to opioid analgesic use. The remaining authors declare that the research was conducted in the absence of any commercial or financial relationships that could be construed as a potential conflict of interest.

## Publisher's Note

All claims expressed in this article are solely those of the authors and do not necessarily represent those of their affiliated organizations, or those of the publisher, the editors and the reviewers. Any product that may be evaluated in this article, or claim that may be made by its manufacturer, is not guaranteed or endorsed by the publisher.

## Footnotes

^1^Scherer EA, Kim SJ, Metcalf SA, Sweeney MA, Wu J, Xie H, et al. The development and validation of a momentary self-regulation scale. *JMIR Mental Health* (under review).
